# Characterisation of the bacterial and archaeal microbiota in processed colostrum collected from a spring-calving dairy herd

**DOI:** 10.1371/journal.pone.0353693

**Published:** 2026-07-22

**Authors:** Sabine Scully, Bernadette Earley, Paul E. Smith, Matthew S. J. Finnie, Catherine McAloon, David A. Kenny, Sinéad M. Waters

**Affiliations:** 1 Animal and Bioscience Research Department, Animal and Grassland Research and Innovation Centre, Teagasc Grange, Dunsany, Co. Meath, Ireland; 2 School of Veterinary Medicine, University College Dublin, Belfield, Dublin, Ireland; Public Library of Science, UNITED KINGDOM OF GREAT BRITAIN AND NORTHERN IRELAND

## Abstract

Colostrum feeding is critical for neonatal calf health, providing immunoglobulins (Ig) and other bioactive compounds that support immune function and early microbiome development. While the microbiota of fresh colostrum has been characterised, colostrum on commercial dairy farms is often refrigerated and reheated prior to feeding – practices that may alter its microbial composition. Therefore, the objective of this study was to characterise the prokaryotic community of refrigerated and reheated (processed) colostrum collected immediately before calf feeding. Twenty-one processed colostrum samples were collected from a single, primi- and multiparous Holstein-Friesian and Jersey, spring-calving dairy herd with no more than two donors contributing to each sample. Colostrum samples were refrigerated for no more than 24h and then re-heated in a 38°C water bath for 60 minutes. Colostrum samples were collected immediately prior to being fed to the calf. Microbial DNA was extracted and16S rRNA gene amplicon libraries were sequenced using the Illumina platform. Raw sequencing data were processed in *R* via the *DADA2* pipeline, and an amplicon sequence variant (ASV) table was generated. Taxonomy was assigned using the SILVA database (v. 138.1) and data were subjected to α- and β-diversity analysis using *Phyloseq, Microbiome* and *Vegan*. Breed and parity had no effect (P ≥ 0.05) on α- and β-diversity. The mean Shannon index score (α-diversity) was 2.26 (SE 0.18), indicating unevenness and low levels of richness. Microbial community composition varied considerably between samples. Five archaeal ASV genus groups were identified, with *Methanobrevibacter* dominating this community(relative abundance (RA) of 85.19%). Four bacterial phyla were identified as the major contributors to the bacterial component of processed colostrum. Only 39 ASV genus groups were identified as having a RA > 0.05%. Processed colostrum was dominated by *Pseudomonas* (RA = 20.97%) and *Acinetobacter* (RA = 18.65%). These genera, along with 11 others, including *Romboutsia, Flavobacterium. Lachnospiraceae NK3A20* group and *Clostridium sensu stricto 1* were present across all samples and thus considered core bacteria. Overall, these findings indicate that refrigeration and reheating may significantly alter the natural colostrum microbiota, reduce diversity and increase heterogeneity of the community composition. Further research is needed to determine how these changes influence microbial seeding and calf health outcomes.

## 1. Introduction

The importance of colostrum to the neonatal calf is well known and described in detail by others, including Geiger [1], Silva et al [2] and Baumrucker et al [3]. Colostrum acts as a mechanism of transfer for immunoglobulins (Igs) from the dam to the calf, as this does not occur *in utero* in ungulates [3]. There is growing interest in understanding colostrum as a biological matrix. Recent research has begun to investigate additional bioactive components of colostrum and their roles in neonatal calf health and development [4–6]. Colostrum collection, storage and processing is common in the dairy industry – however some of these practices have significant impacts on these bioactive compounds. Chandler et al. [4] recently investigated how leukocytes and microRNAs vary among fresh, heat-treated and frozen colostrum. They found that both heat-treating and freezing colostrum after collection eliminated viable leukocytes and altered microRNA abundance. Additionally, heat-treatment above 60°C reduces Ig G concentrations in colostrum [7]. It also leads to reduced Ig A, insulin and insulin like growth factor 1 concentrations [8]. Regardless of these reductions, heat-treatment of stored colostrum is still recommended to eliminate bacteria and prevent disease [9]. Heat-treating colostrum successfully eliminates pathogenic microorganisms that may contaminate it during collection and storage [10]. However, because colostrum is a source for pioneering microorganisms for the calf [11–14], there is increasing concern that colostrum storage and management practices, such as refrigeration and heat-treatment, may alter the natural colostrum microbiota present when consumed fresh. Scully et al. [15] described in detail the prokaryotic community observed in fresh colostrum, which was collected directly from the dam and fed immediately to the calf. The microbiota was diverse in community membership and homogenous in community structure. These community members were metabolically versatile, and many are known bovine gut microbiota commensals. In the dairy industry it is common practice to collect colostrum, store it, pool from multiple cows, and reheat before being fed to calves. These practices can significantly alter the microbial community in colostrum, as microorganisms are highly sensitive to environmental changes. Understanding how these management practices alter the colostrum microbiota is essential for optimizing feeding strategies and supporting early-life calf health. While the microbial composition of fresh colostrum has been characterized [15], the effects of storage and reheating remain poorly understood. To address this knowledge gap, this study focuses on characterising the prokaryotic community structure of refrigerated and reheated (processed) colostrum.

## 2. Materials and methods

All materials and methods have been previously described by Scully et al. [15]. All samples were collected during the same trial period and underwent laboratory analysis at the same time as the fresh colostrum samples. However, refrigerated and reheated colostrum samples, referred to as processed colostrum from this point on, underwent commonly practiced commercial dairy farm colostrum storage procedures, which are described below.

### 2.1 Ethics statement

The experiment was undertaken at the Dairygold Research Farm in Kilworth, Co. Cork, Ireland (Teagasc, Animal and Grassland Research and Innovation Centre, Moorepark, Fermoy, Co. Cork, Ireland; 52°09’N; 8d16’W). All animal procedures used in this study were approved by the Teagasc Animal Ethics Committee, were undertaken by trained personnel and are consistent with the experimental license (AE19132/P148) issued by the Irish Health Products Regulatory Authority under European Union legislation (Directive 2010/63/EU) for the protection of animals used for scientific purposes.

### 2.2. Study design and animal model

#### 2.2.1. Donors and management.

Colostrum was sourced from a single, spring-calving primiparous (n = 11) and multiparous (n = 20; 1–10 lactations, mean lactation 2.6 (SE 0.37)) herd consisting of Holstein-Friesian (HO; n = 17) and Jersey (JE; n = 14) cows (Table 1). Thirty-one donors contributed to the 21 processed colostrum samples collected for this study. Ten samples had a total of two colostrum donors, nine samples belonged to a single donor, and two samples had no donor information recorded.

**Table 1:** Sample population frequencies by breed and parity.

Sample population frequencies by breed and parity				
		Primiparous	Multiparous	Total
2 donors (n = 10)	Holstein-Friesian	5	7	12
	Jersey	4	6	10
1 Donor (n = 9)	Holstein-Friesian	2	3	5
	Jersey	0	4	4
No Records (n = 2)	Unknown	Max. 2	Max. 2	2
Total number of donors (n = 21)		11	20	31

**Fig 1:**
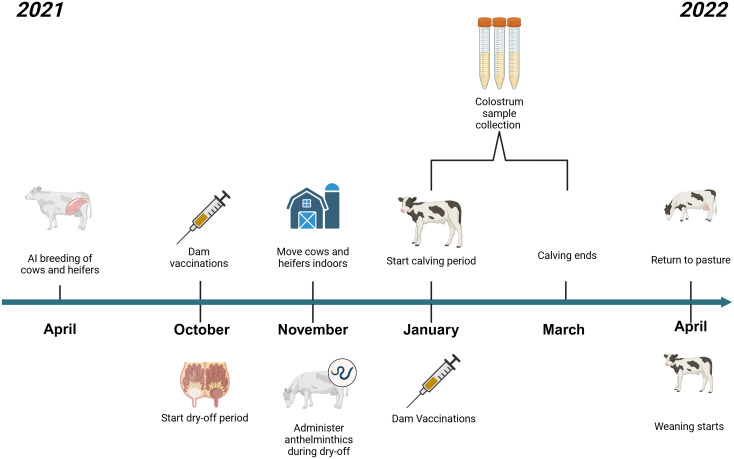
Dam management and sample collection during trial period. Created in BioRender. Scully, S. (2026) https://BioRender.com/6bggcl8.

Dam management has previously been described by Scully et al. [15]. Briefly, cows and heifers grazed perennial rye grass/white clover pastures until November, after which they were group housed until after calving, weather permitting. Pregnant heifers and cows were housed within the same unit, in a free-stall system with slatted floors, automated waste removal and rubber mattresses. When indoors, all cows and heifers received grass silage *ad libitum* with no concentrate supplementation. All in-calf cows and heifers were vaccinated according to the standard operating protocols at the Dairygold Research farm, in line with commercial vaccination programs. At dry-off, cows and heifers were treated for internal and external parasites (Cydectin: 0.5% w/v pour-on for cattle, Zoetis Belgium S.A., Dublin, Ireland; Tribex 10%: Triclabendazole, Channelle Animal Health LTD, Liverpool, England). All multiparous cows had teat sealant (Boviseal, Zoetis Belgium S.A., Dublin, Ireland) applied at dry-off. Eleven of the 18 multiparous cows had somatic cell counts greater than 100,000 and thus received an intramammary antibiotic (Ceravin Dry Cow 250 mg intramammary suspension, MSD Animal Health, Ireland) prior to teat sealing. Primiparous animals (heifers entering first calving and first lactation) received no teat sealant nor udder treatment prior to calving. Using anticipated calving date and clinical signs, pregnant cows and heifers were removed from the main herd and housed in a separate calving unit approximately one week prior to parturition. After removal from the main herd, in-calf cows and heifers were housed together as a group in in the calving shed with deep straw-bedded concrete floors and facilities to isolate individual cows at time of calving if intervention was required. Cows and heifers were provided water and grass silage *ad libitum* and no concentrate supplementation (Fig 1).

#### 2.2.2. Sample and data collection.

Colostrum samples were collected from each mammary gland quarter of individual cows during the spring 2022 calving season (January – March) by research personnel (Fig 1). Colostrum was milked within 2-6h of parturition using a portable milking unit (MK100 034904 2017). Detailed sample collection procedures have been described by Scully et al. [15]. All sampling equipment was cleaned and sterilized and samples were collected in line with best practices for microbiome sample collection [15]. Briefly, milking equipment was cleaned with antimicrobial soap and then rinsed thoroughly with boiling water (100°C) before and after each collection. The teat-cluster was sprayed with 70% ethanol/molecular-grade water solution and wiped with clean kitchen roll after washing and prior to administration to the colostrum donors teat. Immediately prior to milking each teat was wiped clean, individual quadrants of the udder were stripped of its initial contents, teats were then sprayed with 70% ethanol/molecular-grade water solution and wiped clean prior to application of the teat-cluster. All collection, storage and feeding equipment were cleaned with boiling water and antimicrobial soap and then disinfected with 70% ethanol/molecular-grade water solution and wiped dry.

After milking, colostrum was stored in a refrigerator at 4°C for no more than 24h and was reheated to 38°C in a water bath for 60 min prior to feeding to the calf. Colostrum was collected from a single donor and stored individually in clean, sterile buckets (1 bucket = 1 donor) in the refrigerator. Calves (n = 21) were fed colostrum at a volume equivalent to 8.5% of their birthweight in litres, within 2h of birth. In accordance with the farm’s standard operating procedures, and common practice in the dairy industry, calves did not receive colostrum from their own dam. Calves were fed refrigerated colostrum in the order in which it had been collected (oldest to newest). During preparation of the calf’s first feed, colostrum was combined with that from a second donor when necessary. Colostrum quality was tested, after refrigeration and pooling, but before pre-heating and feeding to the calf, using a digital brix refractometer (≤22%; Hanna Digital Refractometer for Sugar, HI96811). Samples used for microbial analysis were collected immediately before calf feeding. For each sample, 30 millilitres (mL) of colostrum was collected aseptically using sterile gloves and aliquoted into three sterile 10mL tube. Aliquots were immediately snap frozen in liquid nitrogen and stored at −80°C until further analysis.

### 2.3. Sample processing

#### 2.3.1. Preparation.

Colostrum samples were thawed gradually over a two-day period, where samples were moved from −80°C to −20°C for a 24h period and then to +4°C for a 24h period [15]. Prior to centrifugation, samples were mixed gently and dispensed into sterile 10mL aliquots. To each aliquot, 600µl of 0.5 EDTA (Tris EDTA buffer, for molecular biology, DNAse, RNAse, Protease free, pH8, Thermo Fisher Scientific, Belgium) was added. Samples were then vortexed to ensure complete mixing and centrifuged at 4500 × g for 20 minutes at 4°C. After centrifugation, the fat layer was removed and discarded, six millilitres of supernatant (fat-free colostrum) was pipetted into three Eppendorf tubes (3 × 2mL). The supernatant was then stored at −80°C until single radial immunodiffusion (sRID) assays were performed. The colostral microbial pellet produced by centrifugation was transferred via pipette to an Eppendorf and stored at −80°C until microbial DNA extraction was performed.

#### 2.3.2. Single radial immunodiffusion.

Single radial immunodiffusion assays were performed to quantify colostrum immunoglobulin A, G and M concentrations. Colostrum Ig concentrations were then used to determine colostrum quality using methodology described by Scully et al. [16]. Briefly, commercially available sRID kits (Triple J Farms, Bellingham, Washington, USA) were used. Three standard controls, supplied with the kit, were applied to plates in a volume of 5 µL each. Colostrum supernatant samples were removed from the −80°C and brought to room temperature (22°C) one hour prior to performing the sRID assay. Five µL of diluted colostrum (1:6 0.9% NaCl dilution) was applied the sRID plates.

#### 2.3.3. Microbial DNA extraction.

Microbial DNA was extracted from the colostral microbial pellet via repeated bead beating and column purification using the Qiagen DNeasy® PowerSoil® Pro Kit (Qiagen, Manchester, United Kingdom) as described by Scully et al. [15]. A negative (blank) extraction was performed for each batch of extractions (n = 6) and subjected to the same extraction procedure as the microbial pellet. Microbial DNA extractions were also performed on the ZymoBIOMICS™ Microbial Community Standard (MC; Zymo Research Corp., Irvine, California, USA), for each extraction kit (n = 3), as an internal positive control. DNA quality was assessed on 0.8% agarose gels with the concentration of extracted DNA quantified on the Nanodrop 1000 spectrophotometer.

#### 2.3.4. Qualitative polymerase chain reaction.

Microbial DNA extracted from colostrum were submitted to qPCR analysis to quantify the presence of bacterial DNA prior to 16S rRNA gene amplicon sequencing, following the methodology described by Kittelmann et al. [17]. The qPCR reaction was performed on an ABI7500 FAST qPCR machine (Applied Biosystems, UK) with 7500 Fast Software v2.3. Reaction conditions were 95°C for 20 seconds, then 40 cycles of 95°C for 3 seconds and 60°C for 30 seconds. After completion of the qPCR run, the cycle threshold was set to 0.2 and baseline was set to automatic then CQ values were calculated. These CQ values were then used to verify the presence of bacteria within the colostrum samples selected for 16S rRNA gene amplicon sequencing. The mean CQ value for the overall dataset was 19.79 (SE 0.92) with a range of 12.27–27.03. The mean CQ value for negative controls was 36.73 (SE 0.16), whereas mean CQ value for positive *Staphylococcus* controls was 12.30 (SE 0.15). Two negative controls returned as ‘undetermined’. The CQ values obtained from processed colostrum, attained from qPCR, confirmed that, although the samples were low in biomass, sufficient bacteria were present for sequencing.

#### 2.3.5. Sequencing.

Extracted DNA was sent to Macrogen (Seoul, South Korea) for 16S rRNA gene amplicon library preparation and sequencing. Samples underwent PCR amplification, targeting the V4 hypervariable region of the 16S rRNA gene, using 515F/806R primers [18] designed with Nextera overhang adapters and Herculase II Fusion DNA Polymerase (Agilent, Santa Clara, California, USA). Cycle conditions were as follows: 95°C for 3 min, 25 cycles at 95°C for 30 s, 55°C for 30 s, 72°C for 30 s, and then 72°C for 10 min. PCR amplicon purification was performed using standard AMpure paramagnetic bead protocol (Beckman Coulter, Indianapolis, Indiana, USA). Amplicons were pooled together in equal concentration and subjected to sequencing on the Illumina MiSeq using the 500-cycle version 2 MiSeq reagents kit (Illumina, San Diego, California, USA) on one flow cell.

### 2.4. Statistical and sequencing analysis

All data were subjected to statistical and sequencing analyses as described by Scully et al. [15]. Briefly, animal data used for statistical analysis were analysed using SAS software (Version 9.4, SAS institute Inc., Cary, NC, USA). Data were checked for normality and homogeneity of variance (PROC UNIVARIATE procedure) and then subjected to ANOVA (PROC MIXED). The model included fixed effects of breed, parity and their interactions. Mean values were considered statistically significant at P ≤ 0.05. Non-significant terms (P > 0.05) were excluded from the final model. Parity of samples where colostrum was sourced from two donors was determined by calculating the mean lactation number of both donors.

Sequencing data were initially processed using *DADA2* (v. 1.26.0) and submitted to the pipeline as described by Callahan et al. [19] following a similar methodology described by Smith et al. [20]. Data underwent quality control, filtering, trimming after which an Amplicon Sequence Variant (ASV) table was constructed, and chimeric sequences were removed. Taxonomy was assigned to sequence variants using the SILVA database (v. 138.1) and phyla names were updated according to Oren and Garrity [21]. Sample metadata, sequence taxonomy, and ASVs were combined into a phyloseq object using *Phyloseq* (v. 1.42.0) [22]. Based on plateauing of the rarefaction curve, sequencing was conducted to a sufficient depth and data was not rarefied. Identification and removal of contaminants was performed with a threshold of 0.5 using *decontam* (v. 1.22.0) [23]. After removal of contaminants, data were separated by bacteria and archaea and analysed independently. Subsequently, alpha (α; Shannon) diversity was calculated for each sample. For comparisons of beta (β; composition) diversity and differential abundance analysis, the relative abundance (RA) of ASVs were calculated, and those that were not present in >0.05% were removed before further analysis. Beta diversity was depicted graphically using non-metric multidimensional scaling (NMDS) using Bray-Curtis plots. Due to low diversity and abundance, analysis of archaea was unable to proceed beyond taxonomic classification and α-diversity analysis.

For downstream sequencing analysis, colostrum samples were classified as primi- or multiparous based on the mean lactation number calculated using the lactation of the donor(s) that contributed to the sample. Prior to assessing the effect of breed, parity, and their interactions on overall prokaryotic community structure, the homogeneity of group dispersions was assessed between groups. Following this, PERMANOVA tests based on Bray-Curtis dissimilarities, 9,999 permutations and a significance level of P < 0.05 were implemented to determine if breed or parity affected prokaryotic community structure. Both assessment of homogeneity of group dispersions and PERMANOVA were conducted using *Vegan* (v. 2.6.4) [24]. Core bacteria identification and other analyses were performed using *Microbiome* [25], post-filtering for a RA of >0.05%. The core bacteria were defined as those taxa agglomerated at the genus level shared across colostrum samples [26]. All analysis was performed at the phylum and genus level due to poor species level classification. A spearman’s rank-order correlation for non-parametric data was run between core bacterial ASV groups and colostrum quality data to explore potential relationships between core bacteria, colostrum quality and the two. Correlations (effect size and strength of the correlation, denoted as r) were described using the following: 0.00–0.19, “very weak”; 0.20–0.39, “weak”; 0.40–0.59, “moderate”; 0.60–0.79, “strong; and 0.80-1.00, “very strong” [27]. Only correlations with a P ≤ 0.05 were considered statistically significant.

## 3. Results

### 3.1. Colostrum quality

The median number of colostrum donors per sample collected was two. Mean lactation number of donors was 2.6 (SE 0.37). The coefficient of determination (r^2^) values for sRID plate analysis ranged from 0.97–1.00. No effect (P > 0.05) of breed or parity or their interaction were observed on mean colostral Ig A, Ig G, or Ig M concentrations (Table 2). However, there were differences in colostral Ig G concentration, where colostrum sourced from primiparous donors had + 2.54 mg/mL more total Ig G than multiparous donors. In contrast, concentrations of Ig A and Ig M were higher in colostrum from multiparous donors, by 3.44 mg/mL and 1.23 mg/mL, respectively. There were breed differences, where colostrum sourced from Holsteins had lower Ig concentrations than Mixed (HO + JE; Ig A: + 1.99; Ig G: + 25.28; Ig M: + 2.89 mg/mL) and lower Ig A and Ig M concentrations than Jersey (+1.84, + 1.04 mg/mL, respectively). Both Mixed (+35.58 mg/mL) and Holstein (+10.30 mg/mL) had greater total Ig G concentrations than Jerseys. The breed and parity differences should be interpreted with caution given the small sample sizes per group, which limit statistical power and increase the likelihood that observed trends may reflect random variation rather than true biological effects.

**Table 2:** Mean lactation and colostral immunoglobulin concentrations of processed colostrum samples.

Mean Lactation and Colostral Immunoglobulin Concentrations of Processed Colostrum Samples												
	All^1^		Parity			Breed				P-values		
	Mean	SE^2^	Primi^3^ (n = 5)	Multi^4^ (n = 14)	pooled SE	HO^5^ (n = 8)	JE^6^ (n = 5)	MIX^7^ (n = 6)	pooled SE	Breed	Parity	B*P^8^
Lactation (No.)^9^	2.60	0.37	1.00	3.11	0.21	2.25	3.10	2.50	0.68	--	--	--
Ig^10^ A (mg/mL)	10.66	0.88	8.21	11.65	0.84	9.63	11.47	11.62	1.67	0.79	0.17	0.79
Ig G (mg/mL)	135.98	6.45	139.1	136.56	12.9	131.96	121.66	157.24	11.03	0.19	0.77	0.14
Ig M (mg/mL)	9.29	0.59	8.3	9.53	0.9	8.02	9.06	10.91	0.86	0.29	0.38	0.77

^1^All: all data; ^2^SE: Standard Error; ^3^Primi: Primiparous; ^4^Multi: Multiparous; ^5^HO: Holstein-Friesian; ^6^JE: Jersey; ^7^MIX: colostrum collected from 2 donors of which donors were HO and JE; ^8^B*P: Breed × Parity interaction; ^9^Lactation (No.): mean lactation number of colostrum donor(s); ^10^Ig: Immunoglobulin.

### 3.2. Sequencing and microbial community composition

**Fig 2:**
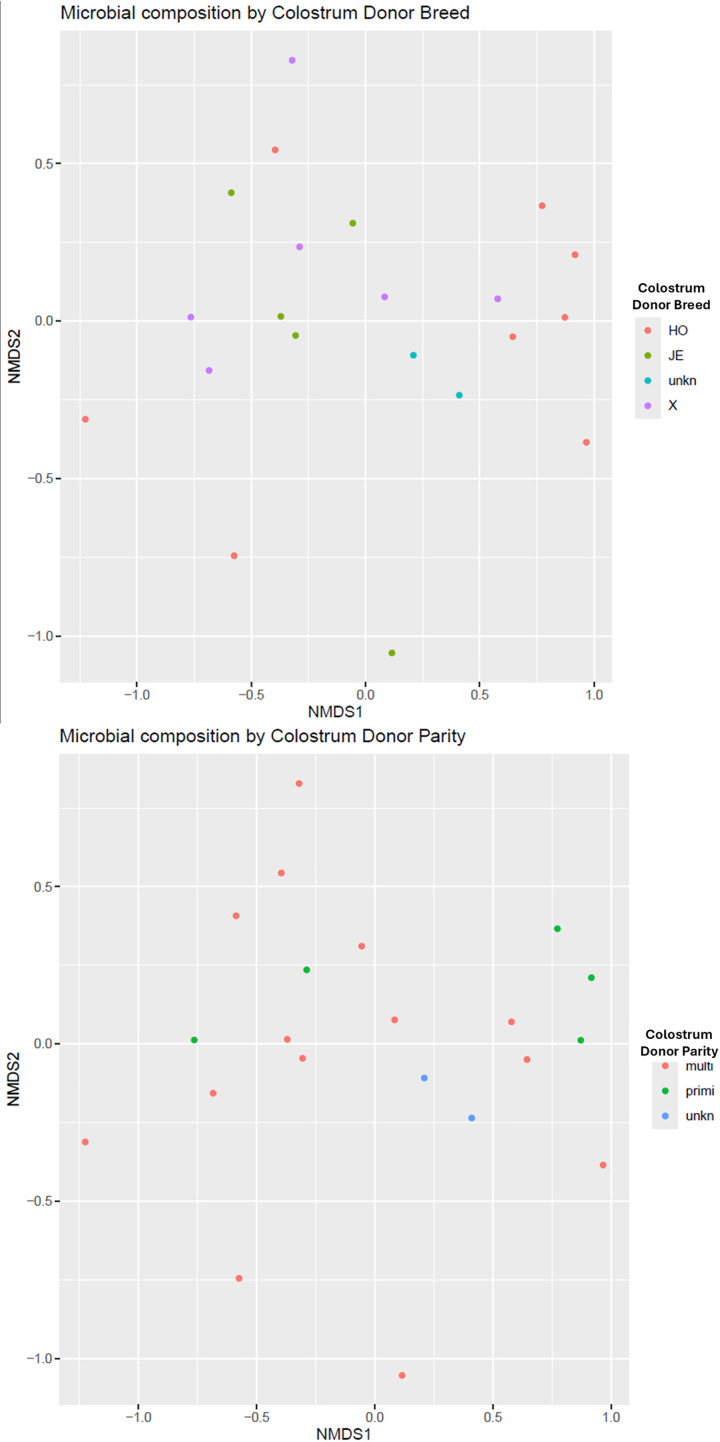
Non-metric Dimensional Scaling plots based on Bray-Curtis dissimilarities and 9,999 permutations to depict microbial community composition by breed (A) and parity (B).

After quality filtering, merging and removal of chimeric sequences, an average of 98,856 (SE 7,112) reads per sample were generated, ranging from 20,153–138,723 reads per sample. This resulted in 6,883 unique ASVs identified at the genus level. Decontamination led to the removal of 2.98% of these ASVs. Post decontamination, ASVs were agglomerated at the genus level, resulting in 446 genus groups. Of these, five ASV genus groups belonged to Archaea. Archaeal analysis did not go beyond taxonomic identification due to low diversity and abundances. The remaining 441 genus groups belonged to Bacteria. Positive controls run for each extraction kit were strongly correlated to one another with r ranging from 0.83–0.95 (P ≤ 0.01). Both breed and parity were found to be homogenous within group during assessment of homogeneity of group dispersions (parity: P = 0.10; breed: P = 0.07). Based on ANOVA results, no effects of breed (P = 0.26) or parity (P = 0.65) or any interaction, was observed on α-diversity (Shannon Index), and mean Shannon index score was 2.26 (SE 0.18) and ranged from 0.92–3.61 (Table 3). Breed (P = 0.28) and parity (P = 0.75) had no effect on β-diversity (community composition) based on PERMANOVA. Across all samples, the prokaryotic community composition, regardless of breed or parity, appears to be heterogenous in nature (Fig 2).

**Table 3:** Mean Shannon index scores measuring evenness and richness to depict α-diversity of prokaryotic microbiota in processed colostrum.

Mean Shannon Index scores measuring alpha diversity of microbiota in processed colostrum														
	All^1^		Parity				Breed					P-values		
Shannon	Mean	SE^2^		PRIMI^3^	MULTI^4^	Pooled SE		HO^5^	JE^6^	MIX^7^	Pooled SE	Breed	Parity	B*P^8^
All Data	2.26	0.18		2.12	2.27	0.35		2.55	2.04	1.98	0.30	0.26	0.65	0.26
Bacteria^9^	2.25	0.17		2.11	2.26	0.34		2.53	2.03	1.97	0.30	0.26	0.65	0.26
Archaea^10^	0.19	0.05		0.23	0.18	0.08		0.21	0.08	0.19	0.30	0.10	0.82	0.21

^1^All: all data; ^2^SE: Standard Error; ^3^PRIMI: Primiparous; ^4^MULTI: Multiparous; ^5^HO: Holstein-Friesian; ^6^JE: Jersey; ^7^MIX: colostrum collected from 2 donors of which donors were HO and JE; ^8^B*P: Breed × Parity interaction; ^9^Bacteria: Shannon Index Scores calculated using only those reads identified as Bacteria; ^10^Archaea: Shannon Index Scores calculated using only those reads identified as Archaea.

### 3.3. Archaea

**Fig 3:**
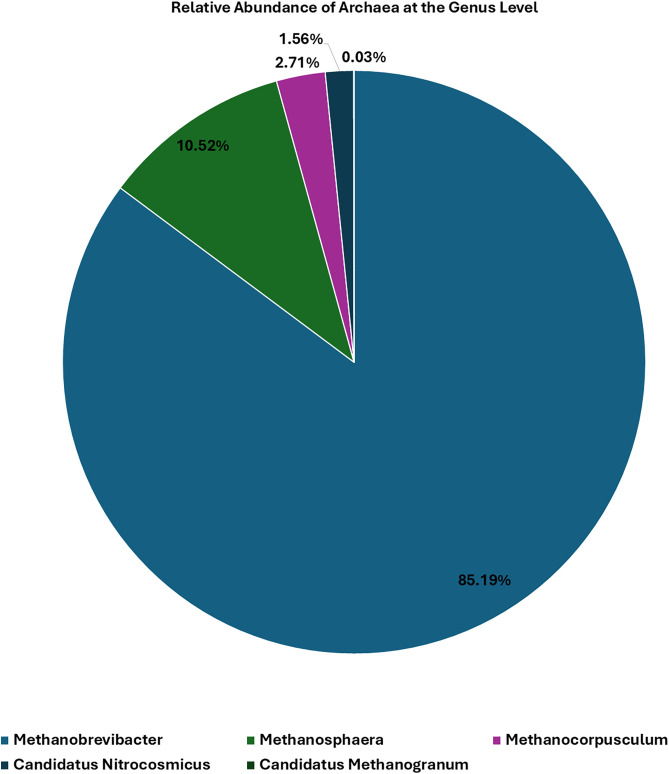
Relative abundance of archaeal genera observed in processed colostrum depicted using a pie chart.

Five archaeal genera were identified (Fig 3), belonging primarily to the phylum *Euryarchaeota* (RA = 98.42%) followed by *Thaumarchaeota* (RA = 1.56%) and *Thermoplasmatota* (RA = 0.03%). *Methanobrevibacter* (RA = 85.19%) and *Methanosphaera* (RA = 10.52%) were the two most proportionally abundant genera. *Methanocorpusculum* (RA = 2.71%), *Candidatus Nitrocomiscus* (RA = 1.56%) and *Candidatus Methanogranum* (RA = 0.03%) were also identified.

### 3.4. Bacteria

**Fig 4:**
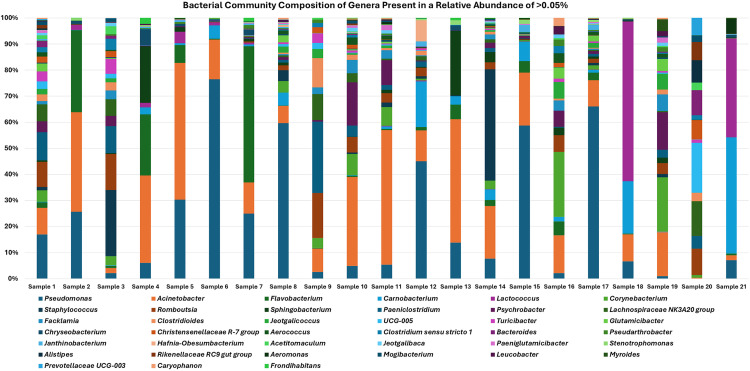
Relative abundances of bacterial genera identified in processed colostrum by sample.

After filtering for a RA of >0.05%, of the 441 bacterial genus groups only 39 were observed to contribute to the bacterial component of the prokaryotic microbiota in processed colostrum (data available on Open Science Framework (OSF)). All bacterial ASV groups belonged to four phyla: *Pseudomonadota* (RA = 43.82%), *Bacillota* (RA = 30.20%), *Bacteroidota* (RA = 11.78%) and *Actinomycetota* (RA = 5.70%). At the genus level, *Pseudomonas* was the most proportionally abundant member of the bacterial community (RA = 20.97%), followed closely by *Acinetobacter* (RA = 18.65%), however, composition varied greatly across samples (Fig 4). *Flavobacterium* (RA = 6.42%), *Staphylococcus* (RA = 3.45%) and *Romboutsia* (RA = 3.25%) also contributed to the 10 most proportionally abundant community members.

Twelve ASV genus groups were identified as contributing to the core bacteria of processed colostrum (Table 4). Seven of these genera were among the ten most proportionally abundant, and an additional three were included within the 20 most proportionally abundant ASV genus groups. The two remaining genera, *Aerococcus* and *Clostridium sensu stricto* 1 were low-abundance (RA < 1.0%) community members.

**Table 4:** Core bacterial genera in processed colostrum, their relative abundances and their metabolism types.

Core bacterial genera of processed colostrum, their relative abundance (RA) and metabolisms					
ASV^1^	Phylum	Family	Genus	RA^2^	Metabolism
ASV 3	*Pseudomonadota*	*Pseudomonadaceae*	*Pseudomonas*	20.97	Aerobe^3^
ASV 5	*Pseudomonadota*	*Moraxellaceae*	*Acinetobacter*	18.65	Aerobe
ASV 14	*Bacteroidota*	*Flavobacteriaceae*	*Flavobacterium*	6.42	Facultative^4^
ASV 15	*Bacillota*	*Carnobacteriaceae*	*Carnobacterium*	5.30	Facultative
ASV 6	*Bacillota*	*Peptostreptococcaceae*	*Romboutsia*	3.25	Anaerobe^5^
ASV 40	*Bacteroidota*	*Sphingobacteriaceae*	*Sphingobacterium*	2.80	Aerobe
ASV 11	*Bacillota*	*Peptostreptococcaceae*	*Paeniclostridium*	2.73	Anaerobe
ASV 28	*Pseudomonadota*	*Moraxellaceae*	*Psychrobacter*	2.64	Aerobe
ASV 21	*Bacillota*	*Lachnospiraceae*	*NK3A20 group*	1.76	Anaerobe
ASV 39	*Bacillota*	*Erysipelotrichaceae*	*Turicibacter*	0.83	Anaerobe
ASV 38	*Bacillota*	*Aerococcaceae*	*Aerococcus*	0.65	Facultative
ASV 61	*Bacillota*	*Clostridiaceae*	*Clostridium sensu stricto 1*	0.60	Anaerobe

^*1*^*ASV: Amplicon Sequence Variant;* ^*2*^*RA: Relative Abundance, expressed as a percentage (%);* ^*3*^*Aerobe: denotes bacteria which are obligate aerobes, requiring oxygen to grow;* ^*4*^*Anaerobe: denotes bacteria which are obligate anaerobes and cannot survive in oxygenated environments;* ^*5*^*Facultative: denotes bacteria that are capable of function in an environment with or without oxygen.*

### 3.5. Correlations

**Fig 5:**
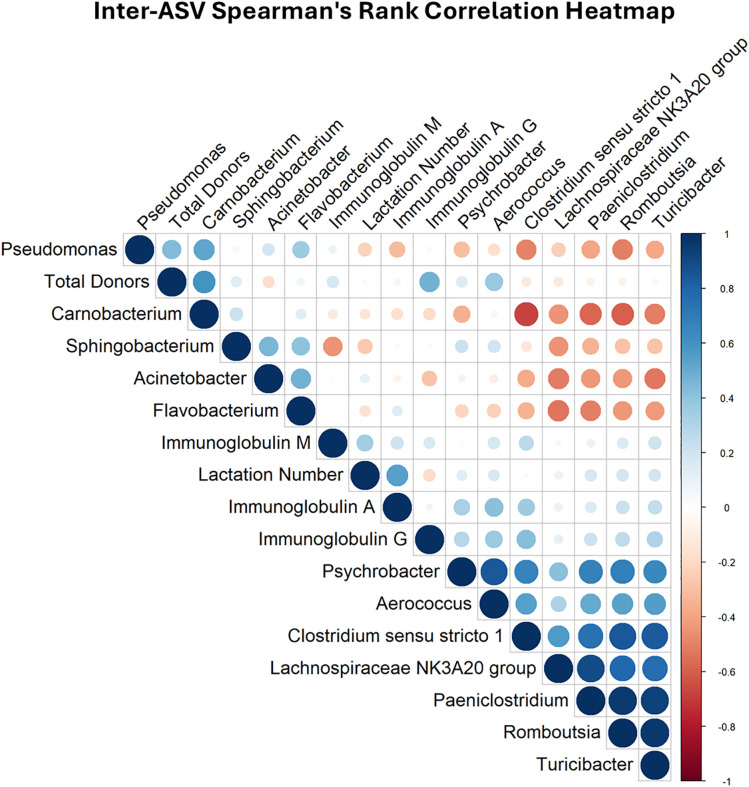
Inter-ASV Spearman’s Rank correlation heatmap.

Several correlations were found between colostrum immunoglobulin concentrations, lactation number and ASV genus groups identified as core bacteria (Table 5; Fig 5). Of these, 25 were moderate, nine were strong and seven were very strong. Mean donor lactation number was moderately correlated to Ig A concentrations (+0.55, P = 0.01). *Sphingobacterium* was moderately correlated to Ig M concentrations (−0.45, P = 0.04) and *Clostridium sensu stricto* 1 was moderately correlated to total Ig G concentrations (+0.43, P = 0.05). Number of colostrum donors per sample was moderately correlated to Total Ig G concentrations (+0.48, P = 0.04) and strongly correlated to *Carnobacterium* (+0.60, P = 0.01). *Carnobacterium* was negatively correlated with *Romboutsia* (−0.60, P = 0.004), *Clostridium sensu stricto* 1 (−0.67, P = 0.001), *Paeniclostridium* (−0.57, P = 0.01), *Lachnospiraceae NK3A20* group (−0.45, P = 0.04) and *Turicibacter* (−0.50, P = 0.02). It was positively correlated with *Pseudomonas* (+0.52, P = 0.05). *Romboutsia* was strongly or very strongly correlated to *Paeniclostridium* (+0.96, P < 0.0001), *Clostridium sensu stricto* 1 (+0.85, P < 0.0001), *Turicibacter* (+0.97, P < 0.0001), *Lachnospiraceae NK3A20* (+0.79, P = 0.0002) and *Psychrobacter* (+0.69, P = 0.001). A variety of other strong or very strong positive correlations were observed between *Psychrobacter, Lachnospiraceae NK3A20* group, *Turicibacter, Aerococcus* and *Clostridium sensu stricto* 1 (data available on OSF). These same ASV genus groups saw moderate negative correlations with *Pseudomonas, Acinetobacter* and *Flavobacterium* (data available on OSF).

**Table 5:** Correlations between colostrum data and microbiota identified as core.

Correlations between colostrum data and microbiota identified as core					
Variable 1	Variable 2	r	p	Direction	classification
*Turicibacter*	*Romboutsia*	0.97	<0.0001	Positive	Very Strong
*Paeniclostridium*	*Romboutsia*	0.96	<0.0001	Positive	Very Strong
*Turicibacter*	*Paeniclostridium*	0.92	<0.0001	Positive	Very Strong
*Lachnospiraceae NK3A20*	*Paeniclostridium*	0.90	<0.0001	Positive	Very Strong
*Clostridium sensu stricto 1*	*Romboutsia*	0.85	<0.0001	Positive	Very Strong
*Aerococcus*	*Psychrobacter*	0.85	<0.0001	Positive	Very Strong
*Clostridium sensu stricto 1*	*Turicibacter*	0.84	<0.0001	Positive	Very Strong
*Lachnospiraceae NK3A20*	*Romboutsia*	0.79	0.0002	Positive	Strong
*Turicibacter*	*Lachnospiraceae NK3A20*	0.77	<0.0001	Positive	Strong
*Clostridium sensu stricto 1*	*Paeniclostridium*	0.75	0.0001	Positive	Strong
*Psychrobacter*	*Romboutsia*	0.69	0.001	Positive	Strong
*Clostridium sensu stricto 1*	*Carnobacterium*	0.67	0.001	Negative	Strong
*Psychrobacter*	*Paeniclostridium*	0.67	0.001	Positive	Strong
*Clostridium sensu stricto 1*	*Psychrobacter*	0.67	0.001	Positive	Strong
*Turicibacter*	*Psychrobacter*	0.64	0.002	Positive	Strong
**Number of lactation donors**	***Carnobacterium***	**0.60**	**0.01**	**Positive**	**Strong**
*Romboutsia*	*Carnobacterium*	0.60	0.004	Negative	Strong
*Paeniclostridium*	*Carnobacterium*	0.57	0.01	Negative	Moderate
*Clostridium sensu stricto 1*	*Lachnospiraceae NK3A20*	0.56	0.01	Positive	Moderate
**Lactation Number**	**Colostral Immunoglobulin A**	**0.55**	**0.01**	**Positive**	**Moderate**
*Aerococcus*	*Turicibacter*	0.55	0.01	Positive	Moderate
*Aerococcus*	*Clostridium sensu stricto 1*	0.54	0.01	Positive	Moderate
*Turicibacter*	*Acinetobacter*	0.53	0.01	Negative	Moderate
*Lachnospiraceae NK3A20*	*Flavobacterium*	0.53	0.01	Negative	Moderate
*Aerococcus*	*Romboutsia*	0.53	0.01	Positive	Moderate
*Carnobacterium*	*Pseudomonas*	0.52	0.01	Positive	Moderate
*Lachnospiraceae NK3A20*	*Acinetobacter*	0.51	0.02	Negative	Moderate
*Paeniclostridium*	*Flavobacterium*	0.51	0.02	Negative	Moderate
*Aerococcus*	*Paeniclostridium*	0.51	0.02	Positive	Moderate
*Romboutsia*	*Pseudomonas*	0.50	0.02	Negative	Moderate
*Turicibacter*	*Carnobacterium*	0.50	0.02	Negative	Moderate
*Clostridium sensu stricto 1*	*Pseudomonas*	0.49	0.02	Negative	Moderate
**Number of lactation donors**	**Colostral Immunoglobulin G**	**0.48**	**0.04**	**Positive**	**Moderate**
*Flavobacterium*	*Acinetobacter*	0.48	0.03	Positive	Moderate
***Sphingobacterium***	**Colostral Immunoglobulin M**	**0.45**	**0.04**	**Negative**	**Moderate**
*Sphingobacterium*	*Acinetobacter*	0.45	0.04	Positive	Moderate
*Lachnospiraceae NK3A20*	*Carnobacterium*	0.45	0.04	Negative	Moderate
*Lachnospiraceae NK3A20*	*Sphingobacterium*	0.45	0.04	Negative	Moderate
*Romboutsia*	*Acinetobacter*	0.44	0.05	Negative	Moderate
*Romboutsia*	*Flavobacterium*	0.44	0.05	Negative	Moderate
***Clostridium sensu stricto* 1**	**Colostral Immunoglobulin G**	**0.43**	**0.05**	**Positive**	**Moderate**
*Paeniclostridium*	*Acinetobacter*	0.43	0.05	Negative	Moderate

## 4. Discussion

Processed colostrum was comprised of a limited and highly heterogeneous prokaryotic community. Community structure varied considerably across samples with the archaeal community dominated by *Methanobrevibacter* and *Methanosphaera* while the bacterial community was dominated by *Pseudomonas* and *Acinetobacter*.

### 4.1. Colostrum quality

Previous work has shown that post-calving collection interval results in decreased total Ig G concentrations, thus reducing the quality of colostrum [28,29]. In the present study, processed colostrum was generally collected from cows that calved overnight, thus delaying colostrum collection and allowing for a larger post-calving collection interval. Variations in colostral Ig G concentrations could also be associated with the mixing of colostrum, where combining colostrum from different donors may have led to a dilution of Ig G concentrations, if one was of lesser quality [30]; although this is unlikely as there was a positive relationship between number of colostrum donors and Total Ig G concentrations. While reduced colostral Ig G concentrations can negatively affect calf health; both Barry et al. [31] and King et al. [32] have reported that pooling high-quality colostrum has minimal effect on calf passive immune status. Similarly, Scully et al. [16], found that colostrum source (dam or mixed) had no effect on calf passive immune status or disease incidence.

In the present study, while collected 2-6h after calving, and combined with colostrum from another donor, all colostrum sampled was of excellent quality, where mean Ig G concentrations reported were 2.7 times greater than the minimum threshold of 50 mg/mL required to be considered of adequate quality. Immunoglobulins A and M provide additional immune support to the neonatal calf, particularly at the epithelial surface [33]. High mean Ig A and Ig M measures (10.66 mg/mL and 9.29 mg/mL, respectively) reported in the present study support the assertion that the colostrum sampled is of excellent quality, regardless of storage practices and delayed collection.

Although no significant interaction between breed and parity was detected, trends in immunoglobulin concentrations for parity and breed were reported to provide biological context. The descriptive differences may offer preliminary insights into potential patterns that could be relevant for colostrum quality and calf immunity. However, interpretation should be approached with caution due to the small sample sizes within breed and parity groups, which limit statistical power and increase the likelihood that observed differences reflect random variation rather than true biological effects. Consequently, these findings should be considered exploratory and warrant validation in larger, more balanced studies

### 4.2. Microbial community composition

In the dairy industry, colostrum is commonly processed, refrigerated and reheated, before being fed to the newborn calf. However, storage increases the risk of bacterial contamination and proliferation [34], which can compromise passive transfer of immunity and increase the risk of calfhood disease [35]. The reheating of colostrum is known to reduce disease incidence rates during the pre-weaning period [9]. There is concern that heat-treating colostrum may be detrimental to early gut-colonizers [36] in that, heat-treating not only results in the destruction of pathogens, but also commensal and beneficial microbes [37]. Stewart et al. [10] has previously reported that bacterial counts in colostrum are low when collected directly from the udder, and that storage results in a significant increase in bacterial content. More recent research supports the aforementioned work, showing that storing colostrum can promote the proliferation of bacteria [35,38]. As no in-field measurements (Total Bacterial and Coliform Counts) were performed on the colostrum in the present study, the actual bacterial load of the processed colostrum sampled is unknown. Nevertheless, analysis of α-diversity and microbial community composition suggests that storing colostrum not only allowed for bacterial proliferation but also led to reduced population diversity and decreased homogeneity in community membership. These changes were particularly evident when compared to the microbial community membership of fresh colostrum reported by Scully et al. [15]. Higher bacterial loads and decreased diversity could be a result of environmental contamination [34] in combination with the ability of different microbes to adapt to changing environmental conditions, such as temperature. For example, *Pseudomonas* has been observed in fresh colostrum, collected directly from the dam [15], but it is also highly prevalent in the environment and its increased relative abundance in processed colostrum may be a result of this bacterium’s adaptability as well as environmental contamination of stored colostrum. The characterization of the bacterial component of the colostral microbiota in processed colostrum may reflect the negative effects of common colostrum management practices on the colostral microbiome, however, further work is necessary to understand these dynamics.

The absence of breed effect on the colostral microbial community was also observed in fresh colostrum [15]. van Hese et al. [39] previously reported compositional differences in colostrum collected from beef and dairy cattle, however, these differences were not attributed to breed as colostrum sampled came from two different types of production systems. No breed effect has been observed on microbial community composition in the present study, nor in the previous study on fresh colostrum presented by Scully et al. [15]. In a previous study performed by the same author group, no breed effect was observed on the faecal microbiota in pre-weaned Holstein-Friesian and Jersey homebred calves (born on the farm in which they are raised and kept) of the same farm origin [16]. Additionally, Voland et al. [40] found no difference in the rumen microbiota composition of Holstein and Montbéliarde calves, which were also homebred and originated from the same farm. In the present study, all colostrum samples were collected from the primary milking herd, consisting of homebred Holstein-Friesian and Jersey heifers and cows managed under identical conditions. As suggested by Scully et al. [15–16], the breed effects commonly reported in other studies [41–43] may, in fact be confounded by differences in farm origin.

Fresh colostrum characterised by Scully et al. [15] was richer in diversity within individual sample and more homogenous in community composition across samples. In contrast, the processed colostrum examined in the present study had lower α-diversity and was heterogenous in community membership across samples, indicating large variations in microbial composition. This suggests that common colostrum storage and management practices, such as refrigeration, pooling, and reheating alters the natural colostral bacterial community. A study by Yeoman et al. [12] previously observed that refrigeration affected the abundance of certain bacterial genera in colostrum. Interestingly, Yeoman et al. [12], found no difference in α-diversity measures between fresh and refrigerated colostrum. In contrast, Scully et al. [15] reported a mean Shannon index of 3.33 for fresh colostrum, collected from the same herd during the same calving season as the present study, 1.21 units higher than the mean observed for processed colostrum in the present dataset. Yeoman et al. [12] also found that the relative abundance of *Lactococcus* was greater in fresh colostrum and decreased after refrigeration. However, Scully et al. [15] did not detect *Lactococcus* in the fresh colostrum; and yet in the present study, it was observed to be one of the top ten most proportionally abundant ASV genus groups in processed colostrum. These discrepancies may relate to differences in refrigeration time. Yeoman et al. [12] only refrigerated colostrum for four hours, compared with the 24-hour storage period used in the present study. *Lactococcus* may have been present in low abundance (RA < 0.05%) in the fresh colostrum studied by Scully et al. [15], making it undetectable or unreported. Extended refrigeration could have provided favourable conditions for *Lactococcus* proliferation during storage, which may explain the proportionally greater abundance observed in processed colostrum in the present study.

The two most proportionally abundant archaea, *Methanobrevibacter* and *Methanosphaera*, in the present study have been reported in previous milk and colostrum microbiota studies [13,14; 44,45]. They were also the dominant archaeal genera reported in fresh colostrum [15]. Archaea are strict anaerobes; however, they are present in a variety of ecosystems and perform a wide variety of roles within these ecosystems [46]. They are common members of the bovine gastrointestinal microbiomes [47,48] and are capable of adapting to and living under extreme environmental conditions [46]. This ability to adapt may explain why there were not extreme differences in archaeal community composition between fresh and processed colostrum. Both colostrum sources were dominated by *Methanobrevibacter* and *Methanosphaera*, the most commonly reported archaea in the hindgut of neonatal calves [48]. However, fresh colostrum contained seven archaeal ASV genus groups versus only five observed in processed colostrum.

Further work is required to understand the effect of colostrum management and storage practices on the colostral microbiome and what implications this may have on calf health and development, particularly seeding and colonization of the calf gut. The presence of microbes in colostrum has already been proposed to contribute to calf gastrointestinal microbial seeding by Addis et al. [11], Yeoman et al. [12] and Zhu et al. [13], however, additional work is necessary to understand the function of these microbes, the way they interact with the host and other microbes during seeding and colonization, and the impact this may have on the calf hindgut and pre-weaning health.

### 4.3. Implications on calf health

While the present findings indicate that processing like refrigeration and reheating may alter the colostral microbiota, this study did not assess calf gut microbiota or microbiome development or health outcomes. Therefore, any implications for calf health remain speculative and require further investigation. Colostrum contains a variety of bioactive compounds, in addition to immunoglobulins, that are important to calf health and physiological development. Yang et al. [49] reported that colostrum-fed calves not only had higher Ig G levels than those fed transition or bulk tank milk but also had better intestinal development. Colostrum-fed calves were observed to have longer, wider villi with better crypt depth and greater mucosal thickness. Yang et al. [49] concluded that the higher quality the colostrum, the better and faster the immune and intestinal development of the calf. Another study by Martin et al. [50], examined the effect of feeding fresh and frozen colostrum on gut microbes and inflammation in neonatal calves. Freezing colostrum did not affect immunoglobulin content [51] but did destroy other bioactive compounds [4, 51] and likely impacted the development of the prokaryotic community. Martin et al. [50] found that calves fed fresh colostrum from the dam showed less signs of systemic inflammation, were less likely to be anaemic, and were 1.8 times less likely to require antimicrobial treatments than calves fed pooled, frozen colostrum. These calves were also observed to have lower numbers of bacterial genera associated with diarrhoeal disease in faeces during the first week of life. The findings from these studies, and those of Chandler et al. [4], who reported that storage and preparation practices affect colostral leukocyte and microRNA content, highlight the need for further investigation into colostrum as a biological matrix and the roles its bioactive components play in calf immune function and physiological development.

The bacterial families *Lactobacillaceae* and *Lachnospiraceae* in colostrum and calf faeces have previously been associated with increased serum Ig G concentrations and successful passive transfer of immunity [39,52]. Additionally, *Lachnospiraceae* in fresh colostrum was positively associated with BRIX refractometer scores and had wide range of correlations with other bacteria known to be bovine gut commensals and generally associated with good gut health [15]. In the present study, *Lachnospiraceae* was also present in the core bacteria of processed colostrum, however in proportionally lower abundance than what has been reported in fresh colostrum, and no correlations were observed between *Lachnospiraceae* and colostrum quality. Processed colostrum contained fewer bacterial ASV genus groups typically considered bovine gut commensals. There were, however, some correlations between *Lachnospiraceae* and other bacterial genera that are gut commensals, including *Romboutsia* and *Clostridium sensu stricto* 1. Interestingly, the RA of *Clostridium sensu stricto* 1 was moderately correlated to total Ig G concentrations, where, as one increased so did the other. *Clostridium sensu stricto* 1 has previously been reported in colostrum [45,53] and was also part of the core bacteria in fresh colostrum [15]. Although considered a bovine gut commensal [47], it can also act as an opportunistic pathogen, making this positive association with Ig G and bacteria associated with gut health (*Lachnospiraceae* and *Romboutsia),* unexpected. The RA of *Sphingobacterium* was negatively associated with Ig M concentrations, suggesting that as one increased the other decreased. These relationships are associative and should be interpreted cautiously, as the underlying mechanisms are unclear. *Sphingobacterium* was not reported as core in fresh colostrum [15] and appears to be proportionally greater in processed colostrum. *Sphingobacterium, Acinetobacter* and *Pseudomonas*, all proportionally greater in processed colostrum, have recently been associated with antimicrobial resistance genes in bacteria found in beef feedlot water bowls [54]. The mechanisms behind these relationships are unclear. One possible explanation, proposed in previous literature, is that these bacteria bind to immunoglobulins in colostrum, potentially influencing uptake by the neonatal calf [9]. However, this hypothesis was not tested in the present study. The implications of these relationships on calf health and development are unclear, and further research is needed to clarify the functional roles of these bacteria and their interactions with each other and the host.

Overall, processed colostrum was low in microbial diversity, and composed of multiple bacteria, in large abundances, that behave as opportunistic pathogens and are often associated with increased presence of antimicrobial resistance genes. Firm conclusions on the impact this has on calf health cannot be made, however, it does suggest that the feeding of fresh colostrum immediately after collection may benefit the development of the gastrointestinal microbiomes, in particular that of the hindgut which is intricately linked to calf health, physiological development and nutrient absorption.

## 5. Conclusions

Processed colostrum was low in microbial diversity and highly variable in community composition. The bacterial component of processed colostrum was largely composed of *Pseudomonas* and *Acinetobacter*, both of which are known for propagation of antimicrobial resistance genes. There were fewer bovine gut commensals and less metabolic diversity in bacterial community members. The practice of refrigeration and reheating colostrum appears to have significantly influenced microbial diversity and community composition of colostrum samples. However, this study did not include paired comparisons of fresh and processed colostrum from the same donors, limiting the ability to attribute observed microbial changes solely to storage and reheating practices. Future research should incorporate paired sampling and longitudinal approaches, alongside multi-omics study, to directly assess how colostrum processing influences microbial diversity of the gastrointestinal tract, bioactive components, and their downstream effects on calf health and development. Such studies will help elucidate the interplay between colostral microbiota, bioactive components, and host physiology.
